# T3. METACOGNITIVE BELIEFS IN SEVERE MENTAL DISORDERS

**DOI:** 10.1093/schbul/sby016.279

**Published:** 2018-04-01

**Authors:** Tiril Østefjells, Ingrid Melle, Sofie R Aminoff, Ole A Andreassen, Akiah O Berg, Roger Hagen, Tone Hellvin, Trine V Lagerberg, Rachel Loewy, June U Lystad, Kristin L Romm, Leiv Sandvik, Nasrettin Sönmez, Jan Ivar Røssberg

**Affiliations:** 1 Oslo University Hospital and Institute of Clinical Medicine, University of Oslo; 2 Oslo University Hospital and Institute of Clinical Medicine, University of Oslo, Akershus University Hospital; 3 Oslo University Hospital and Institute of Clinical Medicine; 4 Norwegian University of Science and Technology; 5 University of California, San Francisco; 6 Oslo University Hospital

## Abstract

**Background:**

Affective dysregulation and psychotic experiences or symptoms often co-occur in the general population as well as in bipolar and psychotic disorders, suggesting a complex interplay. Early trauma is hypothesised to be important for the aetiology of both, and individuals with early traumatic experiences often develop disorders characterised by an admixture of affective and psychotic symptoms. Early emotional abuse seems to be particularly relevant for both disorders. Studies of common factors associated with affective dysregulation and psychosis in bipolar and psychotic disorders could help further theoretical understanding and tailor therapeutic interventions. Metacognitive beliefs – beliefs that outline the importance or consequence of thoughts – have been proposed as one possible common factor. Compared to healthy controls, patients with affective or psychotic disorders hold higher levels of metacognitive beliefs that could be maladaptive. Metacognitive beliefs have been linked to affective and/or psychotic diagnoses and symptoms in these disorders, and to early trauma in general. However, little is known about the specific relationships between symptoms of bipolar/psychotic disorders, early emotional abuse, and metacognitive beliefs.

This project had three objectives: (1) to examine the prevalence of metacognitive beliefs in bipolar and psychotic disorders, compared to controls; (2) explore whether illness-related factors were linked to metacognitive beliefs; (3) examine if symptomatic responses (depression or positive symptoms) to early emotional abuse were mediated by metacognitive beliefs.

**Methods:**

Patients with a bipolar or psychotic disorder, and healthy controls, were included through the on-going Thematically Organised Psychosis (TOP) Study in Oslo, Norway. Analyses included t-tests for group comparisons, regression analyses, and regression based mediation pathway analyses where the indirect effects were tested with bootstrapped confidence intervals.

**Results:**

Patients with bipolar or psychotic disorders reported higher levels of metacognitive beliefs compared to controls. Metacognitive beliefs were significantly related to depression for all patients. Higher levels of metacognitive beliefs were also related to illness-factors related to a poorer long-term outcome, specifically an earlier age at onset of affective disorder in bipolar disorders, and poorer premorbid social adjustment in psychotic disorders. Metacognitive beliefs significantly mediated the relationship between early emotional abuse and depression. The combination of metacognitive beliefs and depression significantly mediated the relationship between early emotional abuse and positive symptoms. The mediation models explained a moderate amount of the variance in symptoms (R2 = .21 and .29) compared to direct models of early emotional abuse impacting on symptomatic responses directly (R2 = .04 and .03)

**Discussion:**

Our results show that patients with bipolar or psychotic report higher levels of metacognitive beliefs compared to controls, and that such beliefs relate to current symptoms of depression in both patient groups. Our results also suggest that metacognitive beliefs relate to factors present before or at the onset of illness, which are often linked to a poorer long-term outcome in the disorders. Further, our findings suggest that in regards to early emotional abuse, metacognitive beliefs could play a role in an affective pathway to psychosis. Metacognitive beliefs could thus be relevant treatment targets in regards to depression and positive symptoms in bipolar and psychotic disorders.

